# Mutations in Superoxide Dismutase 1 (Sod1) Linked to Familial Amyotrophic Lateral Sclerosis Can Disrupt High-Affinity Zinc-Binding Promoted by the Copper Chaperone for Sod1 (Ccs)

**DOI:** 10.3390/molecules25051086

**Published:** 2020-02-28

**Authors:** Stefanie D. Boyd, Morgan S. Ullrich, Jenifer S. Calvo, Fatemeh Behnia, Gabriele Meloni, Duane D. Winkler

**Affiliations:** 1 Department of Biological Sciences, The University of Texas at Dallas, 800 W. Campbell Rd., Richardson, TX 75080, USA; sdb074000@utdallas.edu (S.D.B.); Morgan.Ullrich@utdallas.edu (M.S.U.); Fatemeh.Behnia@utdallas.edu (F.B.); 2 Department of Chemistry and Biochemistry, The University of Texas at Dallas, 800 W. Campbell Rd., Richardson, TX 75080, USA; Jenifer.Calvo@utdallas.edu (J.S.C.); gabriele.meloni@utdallas.edu (G.M.)

**Keywords:** Sod1, zinc, Amyotrophic Lateral Sclerosis, Ccs, metallo-chaperone, enzyme maturation

## Abstract

Zinc (II) ions (hereafter simplified as zinc) are important for the structural and functional activity of many proteins. For Cu, Zn superoxide dismutase (Sod1), zinc stabilizes the native structure of each Sod1 monomer, promotes homo-dimerization and plays an important role in activity by “softening” the active site so that copper cycling between Cu(I) and Cu(II) can rapidly occur. Previously, we have reported that binding of Sod1 by its copper chaperone (Ccs) stabilizes a conformation of Sod1 that promotes site-specific high-affinity zinc binding. While there are a multitude of Sod1 mutations linked to the familial form of amyotrophic lateral sclerosis (fALS), characterizations by multiple research groups have been unable to realize strong commonalities among mutants. Here, we examine a set of fALS-linked Sod1 mutations that have been well-characterized and are known to possess variation in their biophysical characteristics. The zinc affinities of these mutants are evaluated here for the first time and then compared with the previously established value for wild-type Sod1 zinc affinity. Ccs does not have the same ability to promote zinc binding to these mutants as it does for the wild-type version of Sod1. Our data provides a deeper look into how (non)productive Sod1 maturation by Ccs may link a diverse set of fALS-Sod1 mutations.

## 1. Introduction

Zinc ions are essential trace metals necessary for life. Zinc plays an important structural role in many proteins needed for cell function [1] and there are families of enzymes that use zinc as a catalytic cofactor [2,3]. However, it is not well understood how proteins acquire zinc. Zinc transporters are responsible for importing the ion into the cell and moving it to intracellular compartments such as the endoplasmic reticulum, golgi or nucleus where many proteins requiring zinc are metallated [4]. It has been hypothesized that zinc ions are acquired from a labile pool that exists within the cell, but no conclusive evidence exists related to an exact mechanism [5,6].

One of the proteins that requires zinc is Cu, Zn superoxide dismutase (Sod1), an antioxidant enzyme constitutively expressed in humans and other aerobic organisms [7]. Sod1 catalyzes the dismutation of superoxide; turning superoxide radical into water and hydrogen peroxide in a two-step “ping-pong” reaction [8]. It is present throughout the cell, but is most abundant in the cytosol [9]. Sod1 must be “activated”, which requires three post-translational modifications: zinc binding, copper insertion and formation of an intramolecular disulfide bond [8,10,11]. We and others have shown strong evidence that the copper chaperone for Sod1 (Ccs) assists Sod1 in obtaining each of these modifications [10,12,13]. 

Zinc plays a dual role in Sod1, as it supports both structural stability and enzyme function [13,14]. The binding of zinc by Sod1 stabilizes a region of the enzyme called the “zinc loop”, which significantly decreases Sod1 misfolding and degradation [15]. Zinc binding also assists the enzymatic proficiency of Sod1. Sod1 binds a copper ion in its active site that cycles between Cu(I) and Cu(II) redox states as Sod1 rids the cell of superoxide anions. When the copper is in a reduced form, it binds three histidine residues (H46, H48, H120), and when it is oxidized it binds four histidines (H46, H48, H120, and H63). Zinc is bound in a nearby site by three histidine residues (H63, H71, H80) and a single aspartate (D83). H63 “bridges” the copper and zinc ions, easing the transition between Cu(I) and Cu(II),thereby increasing enzymatic activity 10-fold [6,16].

Mutations to the gene coding for Sod1 are linked to an inherited form of amyotrophic lateral sclerosis (fALS), a neurodegenerative disease resulting in paralysis and eventual death [17,18]. Mutations in Sod1 are the cause of 20% of fALS cases, where a point mutation in a single copy of the *sod1* gene leads to fALS through a toxic “gain-of-function” [19]. There are a multitude of Sod1 mutations that are known to cause fALS, and upwards of 130 sites in Sod1 are disease related. Aggregates containing Sod1 and Ccs can be found in neural tissue expressing fALS-Sod1 mutants, which often lack correct binding of one or both metals and the disulfide bond [20,21,22,23].

While all of the fALS-Sod1 mutations lead to the same disease and outcome, the rates of disease progression vary widely between mutations [24,25]. A4V is the most common mutation in North America, accounting for 50% of Sod1-linked fALS cases [26]. It is also one of the most aggressive forms of ALS, with average survival persisting less than 2 years after diagnosis [27,28]. A4V Sod1 can reach full maturity (copper and zinc bound with an oxidized disulfide bond (i.e., Cu,Zn-Sod1^SS^)) in the cell [29] and has even been shown to have a stronger affinity for copper than wild-type Sod1 [30]. Interestingly, unlike many other fALS-Sod1 mutations, there is no mouse model for A4V, as transgenic mice do not exhibit any symptoms [31].

G93A is another frequently studied mutation. It was one of the earliest established mouse models for the disease and is well-characterized [32]. In this model, metal-free (apo) disulfide reduced G93A (E,E-G93A^SH^) is the primary component of intracellular aggregates, yet a significant amount of fully metallated G93A is present in the soluble fraction [33]. The G85R Sod1 mutation is a commonly used model as the onset of paralytic symptoms corresponds well with the appearance of Sod1 aggregation bundles in neural cells [34]. This form of Sod1 is consistently disulfide reduced, under metallated and/or mis-metallated inside cells [33,35]. The crystal structure shows a novel zinc-coordinating water molecule present in the zinc loop that is speculated to affect zinc affinity [28,36].

Unlike the previous mutations discussed, H80R Sod1 is a metal-binding mutant. H80 is one of the 4 residues responsible for binding zinc, and its mutation to arginine completely abolishes zinc binding at the zinc loop. However, the crystal structure shows that a zinc ion is coordinated at the adjacent active site in place of copper (Zn,E-H80R^SH^) [37]. This type of mis-metallation is often observed in fALS-Sod1 mutants, even when an intact zinc site is present [38].

**Figure 1:**
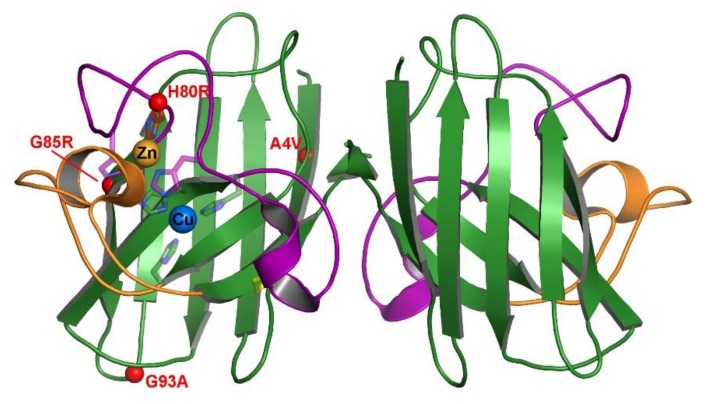
Position of select fALS mutations on mature hSod1. The crystal structure is of wild-type Sod1 (1PU0) with the β-barrel colored green, the electrostatic loop colored orange and the connecting zinc and disulfide loops colored purple. The metal binding residue sidechains are shown as sticks and the metals are shown in their binding sites as labeled spheres. Each of the 4 fALS mutations are shown as small red spheres at their position within the protein structure for visual comparison.

Each of these well-studied mutants has different inherent characteristics and locations within the Sod1 structure (Figure 1); however, all of them lead to the same disease. It is currently unknown what mutual characteristic links these proteins to acquiring fALS symptoms. Additionally, there has lacked clear data showing the effect these mutations have on zinc affinity and the chaperoning actions of Ccs. In this work, we aim to analyze this effect by examining the zinc affinity of these mutations in various stages of maturation. The role of Ccs in facilitating zinc acquisition by Sod1 has been recently published [16]. Here, we further elucidate the changes caused in this interaction by fALS mutations. By providing this missing characteristic, we draw closer to understanding the behavior of Sod1 and any potential role(s) for Ccs in fALS.

## 2. Results

### 2.1. Fluorescent-Labeling of Immature Sod1 Mutants

**Figure 2:**
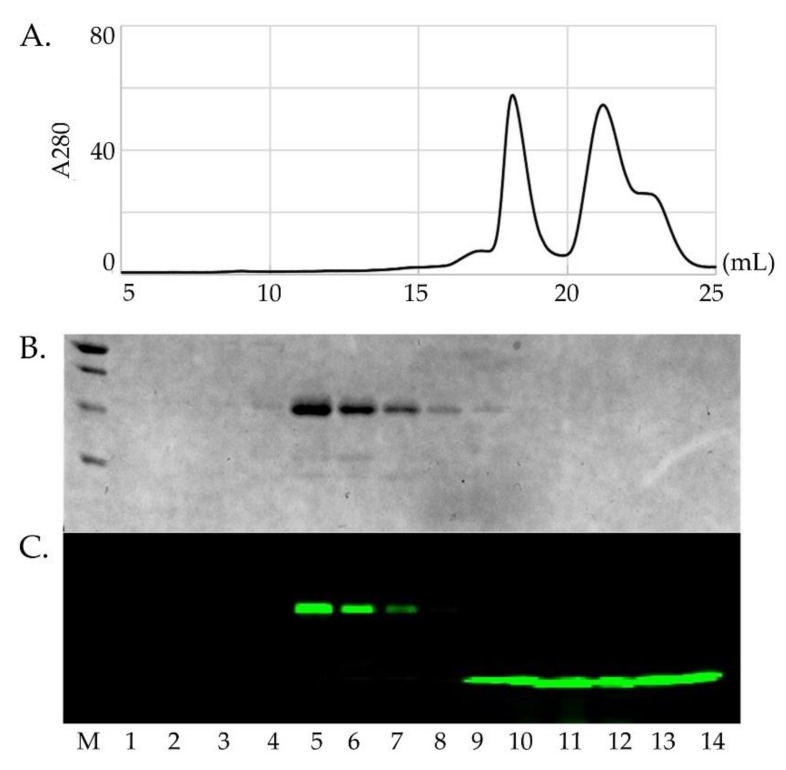
Fluorescent labeling and purification of Sod1. Panel (**A**) shows the purification of an Sod1 protein (left peak, 17–20 mL) after labeling with Alexa_488_ from the remaining free dye (right peak, 20–24 mL). Panel (**B**) shows the SDS-PAGE gel of the peak samples imaged by Coomassie staining. Although the free Alexa_488_ dye shows up in the SEC chromatogram, it does not show up in the Coomassie stained gel. Panel (**C**) shows the same gel that has been imaged using a fluoro-imager set for the emission wavelength for Alexa_488_. This shows that the Sod1 protein (lanes 4–8) has been stably conjugated with the dye and separated from the remaining free dye (lanes 9–14).

Sod1 contains two native cysteine residues involved in the essential intra-subunit disulfide bond (C57-C146). Each fALS-Sod1 variant studied here has an additional C146S background mutation, used to enable specific labeling of Sod1 at position C57 with an Alexa-488 fluorescent dye, which also maintains Sod1 immaturity (i.e., disulfide reduced). Ccs will only bind the reduced form of Sod1 [13]. The labeled proteins were individually purified by size exclusion chromatography in order to separate the Sod1_488_ from unbound label (Figure 2). The fluorescent label is necessary for measuring fine differences in the Sod1•Ccs apparent dissociation constants (K_D_s) as it allows the use of an extremely sensitive detection method (HI-FI) [13,39,40].

### 2.2. Ccs Is Not Prevented from Binding Select fALS-Sod1 Mutants

**Figure 3:**
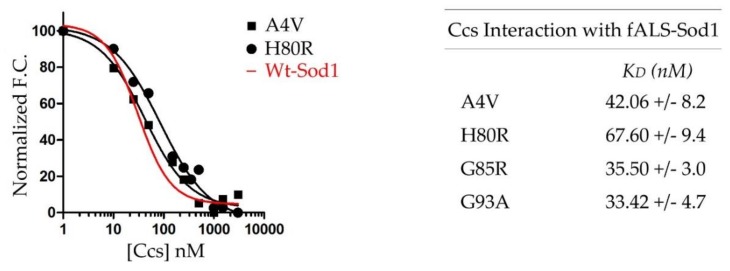
fALS-Sod1 mutants binding with Ccs. The left panel is a combined plot of binding curves for select fALS-Sod1 mutants and Ccs. As shown, the binding curves are comparable to the wT-Sod1 binding curve that we determined previously (red). The right panel shows a list of the fALS-Sod1 mutants that were of focus, here. The K_D_s are all very similar to that of wild-type Sod1 and show that these particular mutations do not hinder Ccs interaction. All binding assays were performed with the human forms of Sod1 and Ccs.

Quantitative binding assays were performed to evaluate differences between fALS-Sod1 interactions with Ccs. The fALS mutants were chosen in an attempt to span the wide variety of metal binding properties (e.g., complete metal binding, mis-metallation, and direct zinc-site disruption) that are seen across the family of pathogenic mutants. Each fALS-Sod1 mutant was kept metal-free (confirmed via ICP-MS) and disulfide reduced (E,E-Sod1^SH^) using the C146S mutation. This results in monomeric Sod1 proteins whose dissociation constants can be compared to previously reported data. The binding affinity between Ccs and fALS E,E-Sod1^SH^ are all extremely tight and within the low nano-molar range, showing that these fALS mutations do not inhibit Ccs binding (Figure 3). The fALS-Sod1•Ccs binding affinities measured: A4V (K_D_ = 42.06 ± 8.2 nM), H80R (K_D_ = 67.60 ± 9.4 nM), G85R (K_D_ = 35.50 ±3.0 nM), and G93A (K_D_ = 33.42 ± 4.7 nM). Previously, we established the affinity of Ccs for wild-type E,E-Sod1^SH^ as K_D_ = 22 ± 6.0 nM [13]. This is the first time that the interaction between Ccs and these fALS-Sod1 mutants have been quantified and shows that their aberrant behavior is not due to a lack of Ccs binding by the immature forms of the proteins.

### 2.3. Ccs Binds Zinc with a High-Affinity

The human form of Ccs binds zinc in its Sod1-like domain 2 (D2) coordinated by residues homologous to those found in the Sod1 β-barrel [8]. We have previously shown that Ccs preferentially binds to a completely immature form of Sod1 (E,E-Sod1^SH^) [13] and others have suggested that Ccs may act directly or indirectly to deliver zinc to immature Sod1 [16,41]. Therefore, we wanted to measure the affinity of the Ccs D2 binding site for zinc and expected that the similar nature of Ccs and Sod1 zinc-binding sites would result in similar affinities. Here, using a TPEN-based zinc-chelation assay, we show that the wild-type Ccs zinc dissociation constant is 2.01 ± 0.34 × 10^−19^ M. This was somewhat surprising as it is ~40-fold tighter than the zinc site in fully mature wild-type Sod1, which was previously measured to be 8.46 ± 2.83 × 10^−18^ M [16]. 

### 2.4. Ccs Promotes Site-Specific Metalation of H80R Sod1

Arginine substitution of the zinc-liganding residue H80 prevents zinc binding at the “zinc-site”. A previously determined crystal structure of H80R (PDB: 3QQD) shows zinc aberrantly bound at the copper/active site and a reduced disulfide bond (Zn,E-H80R^SH^) [37]. Since many ALS-Sod1 patient samples have been reported with this kind of mis-metallation [27,42], we used this mutant to measure the affinity of zinc in the copper site. The immature H80R protein bound zinc with an affinity of 2.78 ± 1.30 × 10^−16^ M (Table 1). This value is ~32-fold weaker than the zinc binding affinity for the “zinc-site” in wild-type Sod1 and over four orders of magnitude less than the affinity for Cu(I) at this site [16]. Notably, zinc loading into the active site was nearly saturated in the samples, with ~94% of the copper/active sites occupied with zinc (measured by ICP-MS after metal reconstitution and exhaustive washing and listed in Table 1). 

We then formed a complex between H80R Sod1 (hSod1) and yeast Ccs (yCcs). If human Ccs was used here, the result would be an average of the Sod1 zinc affinity and the Ccs zinc affinity. Therefore, we utilized the yeast form of Ccs, which does not bind zinc, but is a structural and functional homologue of human Ccs that binds and promotes Sod1 maturation in a nearly indistinguishable manner [10]. The H80R•yCcs complex was purified and then loaded with zinc under the same conditions as the noncomplexed sample described above. The zinc occupancy was a mere 37% and the K_D_ for zinc was measured to be 7.72 ± 1.90 × 10^−16^ M (Table 1). Ccs binding weakens the affinity of the copper/active site held zinc by nearly 3-fold, while dramatically decreasing the total amount of zinc loading into the copper/active site by ~60%. These results suggest that Ccs interaction promotes “site-specific” zinc binding in Sod1 by discouraging mis-metallated zinc binding in the copper/active site [16]. 

### 2.5. Ccs Has Little to No Effect on Zinc Affinity in G93A and G85R Mutations of Sod1

The G93A and G85R mutations are both located on β-strands, but at very different locations within the Sod1 β-barrel. G93 is located at a seemingly innocuous position near a loop between strands 5 and 6 and is commonly termed a “wild-type-like mutant” (WTL). G85, on the other hand, is located near the “zinc-loop” that contains all of the zinc-coordinating residues and is grouped as a “metal-binding region mutant” (MBR) [36]. The zinc affinity of immature G93A was determined to be 2.01 ± 0.19 × 10^−16^ M. The G93A hSod1•yCcs complex zinc affinity was measured 1.36 ± 0.579 × 10^−16^ M. Unlike the H80R mutation, G93A Sod1 can accept copper at the copper site and become fully mature. Therefore, its zinc affinity when fully mature (Cu,Zn-G93A^SS^) was determined to be 6.09 ± 0.01 × 10^−17^ M (Table 1). 

The zinc affinity of immature G85R was 1.11 ± 0.53 × 10^−16^ M. The G85R hSod1•yCcs complex was measured as 3.47 ± 4.10 × 10^−16^ M. Finally, the mature G85R (Cu,Zn-G85A^SS^) zinc affinity was determined to be 3.24 ± 1.18 × 10^−16^ M (Table 1). Ccs binding to G93A and G85R could not promote zinc affinity to the levels of wt-Sod1. However, the zinc occupancy of both Sod1 mutants increased upon Ccs binding (Table 1). All proteins were loaded and extensively washed under the same conditions, then checked for the percentage of zinc bound by ICP-MS. G93A was found to be ~20% loaded, but when bound to Ccs this increased to 73%. Similarly, G85R was 36% loaded when alone and 84% loaded when complexed with Ccs. Zinc binding never exceeded a 1:1 ratio for these mutants, ensuring that extraneous zinc-binding in the copper site is not occurring. Zinc binding will always preferentially bind to an accessible zinc-site due to the several orders of magnitude difference in affinity over the copper site (demonstrated here) and shown in previous structural and biochemical analysis of these mutants, along with A4V, isolated from yeast and reconstituted with metals [28,36]. This suggests that Ccs is providing a beneficial effect on the kinetics of zinc binding by these mutants, although it cannot strengthen their decreased affinities.

### 2.6. A4V Shows Decreased Zinc Affinity with Each Step of Maturation

Similar to G93A and G85R described above, the A4V mutation affects the stability of the β-barrel in Sod1. In a correlated manner, A4V Sod1 also behaves similarly to G93A Sod1 and G85R Sod1 when acquiring zinc. Alone, A4V Sod1 bound zinc at 49% when immature; this increased to 78% when bound by Ccs (both measurements taken after reconstitution and washing). However, when looking at zinc affinity, the A4V mutation sharply contrasts what has been observed for other mutants. The dissociation constant of immature A4V was determined to be 1.91 ± 0.56 × 10^−16^ M for zinc, A4V hSod1•yCcs had an affinity of 3.39 ± 1.04 × 10^−16^ M, and fully mature A4V (Cu,Zn-A4V^SS^) was 1.68 ± 0.10 × 10^−15^ M (Table 1). This shows a decrease in zinc affinity with each incremental step toward maturation. This pattern suggests that the A4V mutation has an inhibitory effect on Sod1 maturation.

### 2.7. Ccs Binding Prevents “wild-Type-Like” Sod1 mutant Oligomerization and Insoluble Aggregation in Vitro

**Figure 4:**
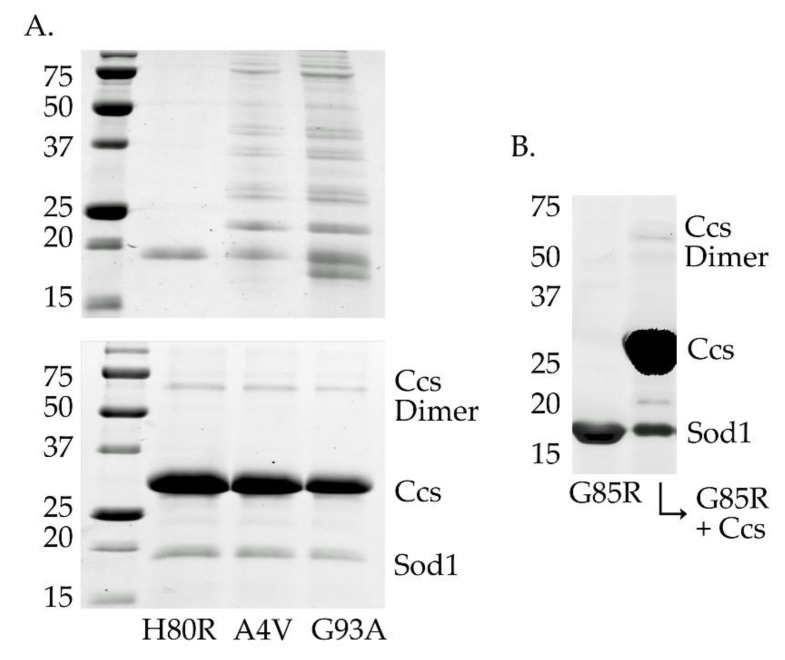
Stabilization of fALS-Sod1 mutants by Ccs. The top gel in panel **A** (the left two gels) compares the stability of two WTL mutants with the metal-binding-region mutant H80R (and G85R in panel **B** (right gel), lane 1), after 4 days at 4 °C. The WTL mutants are highly oligomerized (A4V and G93A lanes) as compared to the two MBR mutants. The bottom gel in panel A shows that the same WTL proteins are protected from severe oligomerization if incubated as a complex with an apo (metal-free) form of Ccs. The complexes shown here are between human Sod1 and human Ccs.

It has previously been reported that A4V and G93A mutations in Sod1 are significantly less stable than wild-type in their immature forms [43]. This same trend does not carry over to many members of the metal-binding region mutants like H80R and G85R. We confirmed early on that, particularly in their disulfide-reduced forms, these two mutations were prone to oligomerization and aggregation within a week after purification, whereas H80R and G85R (and wild-type) Sod1 can remain stable for weeks (Figure 4, panel A, top gel and panel B). Conversely, the A4V and G93A mutations became much less prone to these oligomerization events while in stable complex with an apo (e.g., copper-free) form of Ccs when held under the same conditions and over the same time period (Figure 4, panel A, bottom gel). The use of apo-Ccs is essential to ensure that Ccs cannot perform its activating functions on Sod1 and the interaction is stalled. Ccs confers stability to fALS-Sod1 mutants simply through stable interaction. 

## 3. Discussion

Aberrant zinc binding of fALS-Sod1 mutants has been a topic of much speculation, but little direct evidence for this notion has been provided by the field. In previous work, we showed that wt-Sod1 binds zinc with a very tight affinity compared to other known zinc binding proteins and that interaction Ccs stabilizes site-specific zinc binding by immature Sod1 [16]. To further this work, we have examined how a set of pathogenic fALS mutations directly affect zinc binding by Sod1 and whether or not Ccs interaction can promote high-affinity site-specific zinc binding as observed for the wild-type form of Sod1.

We first wanted to ensure that the cross section of fALS-Sod1 mutants examined could bind Ccs with a similar affinity to the wild-type version of Sod1. All of the fALS mutants tested showed very comparable Ccs binding affinities to what we have previously reported for wild-type Sod1 (K_D_s in the low nano-molar range) [13,16]. However, it would be expected that fALS mutations that occur at the Sod1•Ccs dimeric interface would likely hinder or even eliminate binding, thus preventing any and all Ccs action. 

The human form of Ccs is known to bind zinc at its “Sod1-like” domain 2 and contains matching residues at the same sites as the zincbinding residues in Sod1 [44]. Yeast and other lower eukaryotic forms of Ccs do not contain a complete zinc binding site [12]. Interestingly, Ccs binds zinc with a 100-fold stronger affinity than fully mature wild-type Sod1. It is possible that the adjacent copper-binding domains (D1 and D3) of Ccs, which are not present in Sod1, may play an indirect role in stabilizing zinc-binding in D2. This also argues against Ccs directly supplying zinc to immature Sod1 as metallo-chaperones have been shown to function by simply following an affinity gradient of binding sites between chaperone and target protein binding sites [45].

The fALS-Sod1 mutant H80R does not bind zinc in the canonical zinc site. It does, however, bind zinc in the copper/active site as observed in the crystal structure (PDB: 3QQD) [38]. We use this mutant to examine the zinc affinity in the copper/active site as this and other forms of zinc mis-metallation have been noted in fALS samples [33]. Zinc binding at this site is considerably weaker than the canonical zinc-binding site on Sod1 and Ccs binding further decreased the affinity and occupancy of zinc at this site. This strongly suggests a role for Ccs in guiding proper zinc coordination by Sod1 during the maturation process.

G93A and G85R Sod1 are both β-barrel mutations that are relatively close in sequence, but locations with regard to the zinc-binding site are quite different [28,36]. The G93A mutant structure (PDB: 3GZO) is nearly identical to that of wild-type Sod1 (PDB: 1PU0), from β-barrel conformation, loop placement and metal occupancies. The protein in this crystal structure comes from a small fraction of the total protein has undergone Ccs-mediated maturation and likely misrepresents the majority of the G93A protein expressed in the yeast cells. For instance, it is known that immature G93A (E,E-G93A^SH^) is severely destabilized as compared to wt-Sod1 (common for most WTL fALS mutants) and it is very likely that a large portion of this protein does not reach maturity [20]. The G85R mutant, on the other hand, is proximal to the zinc coordinating residues and zinc loop on Sod1. The crystal structures of G85R (PDBs: 3CQP, 3CQQ, 2VR6-8) display decreased metal occupancy, mis-metallation (zinc in copper site) and disordered loop elements. G85R and many other MBR mutants are quite stable in their immature forms, unlike that of the WTL β-barrel mutants, yet cannot bind metals effectively [36]. Though biophysically very different, both mutants showed a decreased zinc affinity at all stages of maturation when compared to wild-type Sod1 and direct interaction with Ccs was unable to significantly rescue their zinc affinity. However, stable interaction with Ccs did dramatically improve the overall stability and resistance to oligomerization/aggregation for G93A (Figure 4).

A4V is another a β-barrel mutation (located near the Sod1 homodimeric interface) that causes one of the most severe and rapidly progressing forms of fALS [31]. The A4V mutation is not located near the “zinc loop” and does not directly affect its orientation. In fact, the crystal structure of A4V is almost identical to wt-Sod1, with only minor changes throughout the structure (PDB: 1UXM). However, this mutation showed a decreased affinity for zinc that actually worsens with each step of maturation, in direct contradiction to that of wt-Sod1 (Table 1). It has been previously speculated that the conformation of the A4V zinc loop when copper is bound might lower its affinity for zinc, and this data provides further evidence for this idea [46]. Stable interaction with Ccs further weakened the zinc affinity of A4V, but as with G93A, Ccs binding repressed oligomerization events (Figure 4). This mutation seems to actively impede each maturation step, which may serve to increase the time needed to reach full maturity. It is interesting to consider if these unique characteristics of the A4V mutant contribute to how such a seemingly innocuous mutation leads to the rapid disease progression suffered by patients.

fALS-Sod1 mutants have been closely examined and compared by researchers for decades [20,28,37,46,47,48,49]. The mutants examined here were chosen because they are all commonly studied, well-characterized and represent a broad cross-section within the large pool of pathogenic mutants [28,36,37]. We show that the zinc-binding affinities for each mutant are consistently worse than what is observed in wt-Sod1, but the magnitude and pattern of these differences vary by mutation (Table 1). We also show that the zinc occupancy levels are decreased, likely due to varying fractions of these proteins existing as misfolded/off-pathway states commonly observed for these fALS mutants (reviewed in [27,49]). For each fALS-Sod1 mutant possessing a complete zinc-binding site, Ccs binding increased overall zinc occupancy. However, unlike wt-Sod1, zinc binding affinity actually decreased. Ccs binding to immature wt-Sod1 induces a conformation of the disulfide/zinc loop region that promotes high-affinity site-specific zinc binding [10]. Subsequent copper delivery and disulfide bond formation complete Sod1 maturation and secures zinc coordination in a nearly irreversible manner. Mature wt-Sod1 (Cu,Zn-Sod1^SS^) is stable and active under very harsh conditions (e.g., incubation with high concentrations of denaturant and the strong metal ion chelator EDTA) [50,51,52]. This supports the idea, recently described by us and others, that Ccs functions in a molecular-chaperone-like manner where binding alone induces a more stable conformation of Sod1 [10,16,48,53]. This simply may not be true for fALS-Sod1 mutants.

**Figure 5:**
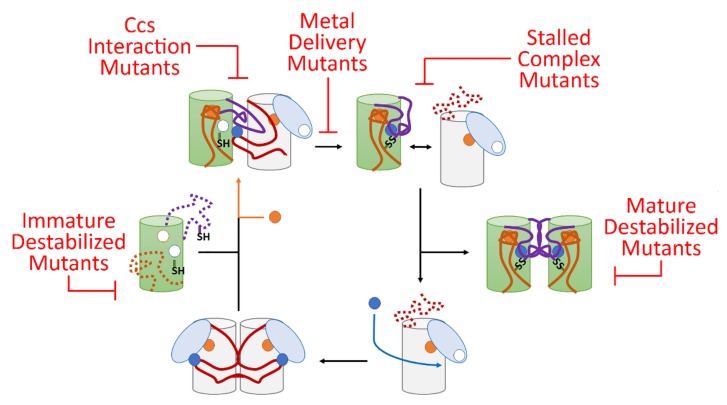
A model for fALS-Sod1 mutations blocking Ccs-mediated Sod1 maturation. In this maturation cycle Sod1 is colored green with the conserved loops colored orange and purple (same as Figure 1). The Ccs molecule is colored by its three domains (D1—light blue, D2—white and D3—red). The copper ions are shown as blue circles and zinc are orange circles. The cycle normally begins as Ccs acquires copper from the cell which stabilizes D3 and homo-dimerization. Recognition and binding to a completely immature (E,E-Sod1^SH^) Sod1 molecule induces site-specific zinc binding in Sod1 followed by Ccs-directed copper delivery and disulfide bond formation. This latter event keys separation of the Sod1•Ccs heterodimer. The mature Sod1 monomer is now free to find another mature Sod1 monomer to form a highly stable Sod1 homodimer that is now a fully active enzyme. The apo-Ccs molecule can now bind another copper ion and enter the maturation cycle again. There are at least five sites within this Ccs-mediated maturation cycle that can be disrupted by fALS mutations and these sites can be populated by a nearly comprehensive list of potential fALS mutant proteins. For example, immature destabilized mutants include L126Z, interaction mutants include I113T, delivery mutants include H80R, stalled complex mutants include C146S, and mature destabilized mutants include A4V, G85R, and G93A.

The inability of Ccs to improve zinc binding status of examined fALS mutants to the levels of the wild-type form provides another point of evidence that the link between the diverse pool of fALS-Sod1 mutants may be an inability to fully complete transaction with Ccs (Figure 5) and [8,27,53]. The argument against this idea has been that Ccs KO mice do not show ALS-like symptoms [54] and Ccs overexpression in mice expressing pathogenic forms of Sod1 actually have earlier symptomatic onset [34]. These data seem to contradict a role for Ccs in fALS pathology. For the former, it is important to note that only a small fraction of Sod1 activity is needed for normal cellular function and the Ccs-independent pathway for Sod1 maturation (found in mammals) can take care of this need. Additionally, the wild-type form of Sod1 is not prone to aggregation in any form [15]. Furthermore, an overabundance of Ccs (overexpression) in the presence of fALS-Sod1 likely exacerbates the problem by forming nonproductive complexes with Sod1 mutants, as previously described [8,10]. The Ccs protein is then likely pulled into the Sod1 laden toxic oligomers. A role for Ccs in Sod1 zinc acquisition is gaining momentum and the concept that fALS-Sod1 mutations deter this function serves to highlight the multifaceted nature of Ccs function and a potential role in fALS-Sod1 pathology. 

Lastly, a large percentage of known fALS-Sod1 mutants can be easily divided into groups that would hinder specific points in the Ccs-mediated Sod1 maturation cycle (Figure 5) and [27]. First, mutants destabilized in their immature state include A4V, G37R, G93A, I113T, L126Z and numerous other WTL mutants. Many of these proteins can reach a full maturity when bound and activated by Ccs, but since Ccs is highly outnumbered by Sod1 in vivo, it is likely that many of these proteins begin the oligomerization/aggregation process before Ccs can do its job. Mutations that preclude direct interaction with Ccs (ex., T54R, I113T, G114A, T116R to name a select few) may be stable in their immature state, but the majority will not ever reach full maturity, since stable Ccs interaction is required for Ccs-mediated maturation, leading to the buildup of immature conformers. Many mutations that directly affect copper binding or delivery have been shown to have stability similar to that of wild-type Sod1 in their immature states [55]. However, these MBR mutants cannot reach maturity due to direct alteration of the metal binding sites in Sod1 (ex., H46R, H48Q, D124V, H80R and many others). Over time this population of immature Sod1 molecules will likely induce pathological effects in the cell. fALS-Sod1 mutations that prevent complete Sod1 maturation produce “stalled complexes” with Ccs where dissociation of the heterodimer does not readily occur. This will include a wide swath of mutants including additional mutants not mentioned in previous groups (ex., C57R, C146R, G85R, and many others in the conserved disulfide loop region). Lastly, there are a group of mutants that can reach maturity, but are severely destabilized in this conformation (ex., A4V, L38V, L84V, G93R and many others) [55]. There is a small fraction of mutants that have characteristics at each step of maturity and activity levels almost identical to wild-type Sod1. Interestingly, some of these mutants happen to fall in established aggregation-forming segments on the Sod1 surface [56]. 

In conclusion, this work provides new insight into zinc binding by fALS-Sod1 mutants and the role of Ccs in Sod1 zinc acquisition. Each of the examined fALS mutants possess a decreased zinc affinity (as compared with wt-Sod1) that could not be rescued by Ccs binding. These novel properties have been acquired via pathogenic fALS mutation and suggest that a role for zinc acquisition in the fALS phenotype deserves closer examination. 

## 4. Materials and Methods

Yeast extract, tryptone, NaCl, BisTris, Tris-base, glycine, β-mercaptoethanol (BME), agar, ammonium persulfate, sodium acetate, acetic acid, EDTA, and TEMED were purchased from Thermo Fisher Scientific (Hampton, NH, USA). DTT, isopropyl 1-thio--d-galactopyranoside, and tris(2-carboxyethyl)phosphine (TCEP) were purchased from GoldBio (St. Louis, MO, USA). Imidazole, Zn_2_SO_4_, and Cu(II)_2_SO_4_, imidazole, monobasic and dibasic sodium phosphate, and acetonitrile were purchased from Sigma-Aldrich (St. Louis, MO, USA). Trichloroethanol was purchased from Acros Organics. Primers for mutagenesis were purchased from Sigma-Aldrich, and the Phusion site-directed mutagenesis kit was from Thermo Fisher Scientific. TPEN was purchased from Tocris Bioscience (Bristol, United Kingdom). Alexa-488 fluorescent dye for labeling was purchased from Life Technologies (Carlsbad, CA, USA). Bacterial strains used were DH5alpha (Invitrogen, St. Louis, MO, USA), BL21 pLysS (DE3) E. coli (Promega, Madison, WI, USA), and XL1-Blue (Stratagene, St. Louis, MO, USA). Chromatography columns were purchased from GE Healthcare (Marlborough, MA, USA). 

### 4.1. Sod1 and Ccs Cloning, Expression and Purification

DNA fragments encoding yCcs1 were generated by PCR from plasmids originally supplied by J. S. Valentine (UCLA). Both yeast Ccs and human Ccs constructs were cloned into a pkA8H vector that contains both contain an inducible LacZ promoter, an 8x-Nterminal His-tag, and a tobacco etch virus (TEV) protease cleavage site and gifted from the Hart lab (UTHSCSA, San Antonio, TX, USA). The A4V and G93A human Sod1 clones were also gifted from the Hart lab. Site specific amino acid changes in Sod1 were done via quick-change mutagenesis to generate G85R and H80R mutants. Sod1 and Ccs1 proteins were expressed in Escherichia coli BL21 (DE3) pLysS. Cells containing these expression plasmids were grown in LB media at 37 °C to an OD600 of 0.6 to 0.8. After induction with IPTG, the cells were transferred to 37 °C for an additional 4 hours before being harvested. Overexpressed proteins were purified using a HisTrap HP Ni2+ affinity column purchased from GE. After purification, the 8x-His-tag was removed from the proteins using TEV protease produced in-house and engineered to contain its own non-cleavable 8X-His-tag. After digestion, the cleaved His-tag and TEV protease are removed from the sample by a final pass through the nickel column. This procedure leaves a two residue (Gly-His) extension on the N-terminus of the purified protein. Sod1•Ccs complexes were purified by size exclusion chromatography. The metal content of purified proteins and protein complexes was determined using inductively-coupled plasma mass spectrometry (ICP-MS), here at UTD (Richardson, TX, USA). Samples for ICP-MS were digested with 1% HNO_3_ for analysis.

### 4.2. Labeling of Sod1 with Fluorescent Labels

The Alexa-488 C5-maleimide dyes (Invitrogen) was mixed at a 1:1 ration with purified apo-C146S Sod1 mutants in a buffer containing 20 mM Tris pH 7.4, 300 mM NaCl and 1 mM TCEP. The mixture incubated in the dark at 4 °C overnight. The micromolar concentration of the protein is kept low to avoid aggregation events. The following morning, the protein was concentrated in spin concentrators to lower the total volume and remove some of the excess dye. The sample was loaded onto a Superdex 200 SEC column from GE. The column was wrapped in foil to keep the sample in the dark during the separation. The sample was fractionated and then run on SDS-PAGE. The excess dye elutes near the bed volume of the column.

### 4.3. Microplate-Based Binding Assays

The preparation and completion of the binding assays were performed as detailed in previous work (13). Binding experiments were done with a reaction buffer containing 20 mM Tris, pH 7.5, 150 mM NaCl, and 1 mM TCEP. The plates were imaged using a GE Typhoon FLA 9500 (UTD, Richardson, TX, USA) scanner using filters specific for the fluorophore’s 488 nm excitation. The binding experiments were completed in replicative quadruplicate on the same plate for comparative and statistical analysis. The fluorescence change was then quantified using Image-Quant TL (UTD, Richardson, TX, USA) and then analyzed, and figures were constructed using GraphPad Prism.

### 4.4. Measuring Zn Affinity of Sod1 Mutants, Sod1 Mutants in Complex with Ccs, and Mature Sod1 Mutants by Equilibrium Dialysis

Proteins and protein complexes were loaded with zinc and washed repeatedly with metal-free buffer to remove any unbound zinc. Complexes were formed using human Sod1 mutants and yeast Ccs, as it does not bind zinc and will not affect affinity calculations. ICP-MS was performed on samples to determine the protein-Zn concentration. Zn-binding affinity experiments were performed at pH 7.4 using a Bel-Art in-line equilibrium cell (1 mL). The dialysis membrane was treated with EDTA and boiled to remove any metal and sulfide contaminants. One side of the chamber for each reaction was filled with a solution of TPEN at 100 μM. The other side contained 10 μM protein–Zn complex and 100 μM TPEN. Excess Sod1 protein that was unbound is considered unable to bind zinc. The zinc-exchange reaction proceeded at room temperature overnight under agitation. ICP-MS was used to analyze the Zn concentrations on both sides. The K_D_ for TPEN at pH 7.4 has previously been determined to be 2.6 × 10^−16^ M [16]. We used this value to determine Zn affinity values. All samples were analyzed in triplicate and from multiple preparations to provide meaningful statistical analysis. Experiments were performed under aerobic conditions and the disulfide bond status was not directly examined in all cases.

### 4.5. Protein Degradation Assay

Purified ALS-Sod1 protein samples were kept at 4 °C for 4 days, then run on a 14% SDS-PAGE gel and visualized with Coomassie stain. ALS-Sod1•Ccs complex samples were kept at 4 °C for 4 days then run on a 14% SDS-PAGE gel and visualized with Coomassie stain.

## Figures and Tables

**Table 1:** Zn(II) dissociation constants and occupancies for fALS-Sod1 mutants.

Immature hSod1	K_D_ (M)	Zn(II) Occupancy
A4V	1.91 ± 0.56 × 10^−16^	49%
H80R	2.78 ± 1.30 × 10^−16^	94%
G85R	1.11 ± 0.53 × 10^−16^	36%
G93A	2.01 ± 0.19 × 10^−16^	20%
hSod1 in complex with yCcs	Fold-Change to measured K_D_	
A4V	~2-fold weaker	78%
H80R	2-3-fold weaker	37%
G85R	No Significant change	84%
G93A	No Significant change	73%
Mature hSod1	Fold-Change to measured K_D_	
A4V	~9-fold weaker	~100%
G85R	No Significant change	~100%
G93A	~3-fold stronger	~100%
